# Protoplast: A Valuable Toolbox to Investigate Plant Stress Perception and Response

**DOI:** 10.3389/fpls.2021.749581

**Published:** 2021-10-05

**Authors:** Guillaume Gilliard, Eloïse Huby, Sylvain Cordelier, Marc Ongena, Sandrine Dhondt-Cordelier, Magali Deleu

**Affiliations:** ^1^Laboratoire de Biophysique Moléculaire aux Interfaces, SFR Condorcet FR CNRS 3417, Gembloux Agro-Bio Tech, Université de Liège, Gembloux, Belgium; ^2^RIBP EA 4707, USC INRAE 1488, SFR Condorcet FR CNRS 3417, Université de Reims Champagne Ardenne, Reims, France; ^3^Microbial Processes and Interactions Laboratory, Terra Teaching and Research Center, SFR Condorcet FR CNRS 3417, Gembloux Agro-Bio Tech, Université de Liège, Gembloux, Belgium

**Keywords:** plant stress response, protein signaling, ion fluxes, cell membrane dynamics, plant protoplasts

## Abstract

Plants are constantly facing abiotic and biotic stresses. To continue to thrive in their environment, they have developed many sophisticated mechanisms to perceive these stresses and provide an appropriate response. There are many ways to study these stress signals in plant, and among them, protoplasts appear to provide a unique experimental system. As plant cells devoid of cell wall, protoplasts allow observations at the individual cell level. They also offer a prime access to the plasma membrane and an original view on the inside of the cell. In this regard, protoplasts are particularly useful to address essential biological questions regarding stress response, such as protein signaling, ion fluxes, ROS production, and plasma membrane dynamics. Here, the tools associated with protoplasts to comprehend plant stress signaling are overviewed and their potential to decipher plant defense mechanisms is discussed.

## Introduction

As sessile organisms, plants are exposed to myriads of potential stresses that can be harmful to their development. These adverse environmental conditions include both biotic and abiotic stresses that increasingly threaten agricultural plant productivity at a worldwide scale. In response, plants have developed an array of mechanisms to survive tough environmental conditions such as drought, heat, cold, nutrient deficiency, pollutants, pathogens, and herbivore attacks. The first crucial step in plant defense is the perception of the stress so that they can respond in a rapid and effective manner (Couto and Zipfel, 2016). While the underlying sensing mechanisms of abiotic stress are not fully elucidated, mostly due to functional redundancy in genes encoding sensor proteins or mutant lethality (Zhu, 2016; Gong et al., 2020), it is believed they are perceived by primary sensory mechanisms (Lamers et al., 2020). Several putative sensors have been ascribed to abiotic stresses perception and are often linked to membrane-associated proteins of the cells, organelles, or nucleus membrane proteins (Zhu, 2016). These sensors will then translate the changing environment into a signaling cascade allowing the plant to coordinate an appropriate response for acclimation. Similarly, plants have evolved an innate immune system to counteract the deleterious effects of biotic stresses (Jones and Dangl, 2006; Saijo and Loo, 2020; Zhou and Zhang, 2020). Once the constitutive plant defenses such as the cuticle, the cell wall (CW) and other physical and biochemical barriers are overrun, the plant plasma membrane (PM) is then at the frontline of stress perception. Through cell surface and intracellular protein receptors, the plant is capable of sensing multiple molecular stress factors, such as MAMPs (microbe-associated molecular patterns), PAMPs (pathogen-associated molecular patterns), and DAMPs (damage-associated molecular patterns), thus initiating a cascade of signal transduction leading to a rapid and effective response from the plant (Cook et al., 2015; Couto and Zipfel, 2016). Both biotic and abiotic stresses share some early signaling events such as the production of reactive oxygen species (ROS) by NADPH oxidases, activation of protein kinases, receptors, or co-receptors through phosphorylation (Kadota et al., 2015; Yu et al., 2017; Zipfel and Oldroyd, 2017; Bigeard and Hirt, 2018), and rapid and transient change of ion fluxes (Jones and Dangl, 2006; Bigeard et al., 2015; Lamers et al., 2020). These fluxes can act on PM potential regulation and activation of Ca^2+^-dependent or K^+^-dependent enzymes (Jeworutzki et al., 2010; Bose et al., 2011; Demidchik, 2014; Wu et al., 2014b; Zipfel and Oldroyd, 2017; Sze and Chanroj, 2018; Yoshioka and Moeder, 2020). Then, activation of transcription factors (TFs) leads to the production of stress-related hormones such as abscisic acid, salicylic acid, jasmonic acid, and ethylene. Upon pathogen attacks, positive and negative crosstalks (Glazebrook, 2005) between these signaling molecules trigger the accumulation of an array of antimicrobial compounds such as pathogenesis-related proteins and phytoalexins (Delaunois et al., 2014).

How plants perceive and respond to these stress signals are essential biological questions and many of them are now investigated through innovative techniques that employ protoplasts as proxy for whole tissue, or even for whole plants. A protoplast refers to a spherical cell whose CW has been removed by digestive enzymes. The first protoplast isolations were developed in bacteria (Weibull, 1953) and fungi (Eddy and Williamson, 1957; Barbara and Bonner, 1959), before being transposed to plants (Cocking, 1960). They are usually obtained from enzymatic digestion of leaf and root tissues or even from cultured cells of a wide variety of species (Fowke et al., 1983; Yoo et al., 2007; Lin et al., 2018; Sangra et al., 2019; Zhao et al., 2019; Cheng and Nakata, 2020). With transformation methods already developed and microscopy techniques fast expending, the protoplast system could ultimately be considered as convenient screening platform to better target future whole plant analyses (Li et al., 2014). Moreover, freshly isolated mesophyll protoplasts are believed to retain the physiological properties of whole plants (Yoo et al., 2007).

**Figure 1:**
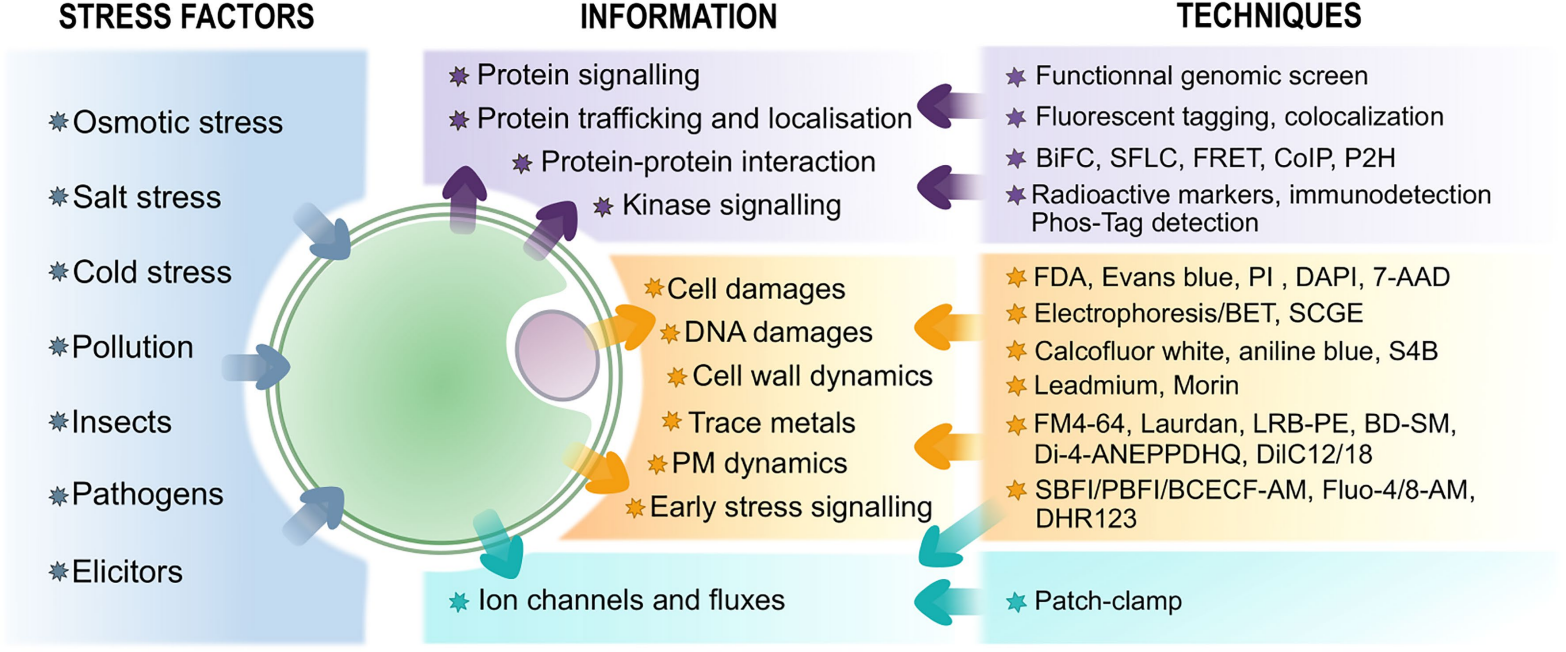
Overview of techniques associated with protoplasts to study plant stress perception and response and the information they provide. The biomolecular-related assays are in purple, fluorescence probes-associated techniques are in orange – see text for further information on the probes, and electrophysiological approaches are in blue.

Protoplasts have already been described as a useful and versatile system to study plant cell reprograming during development (Pasternak et al., 2020) and plastid transformation (Yu et al., 2020). In this review, we will focus on the different approaches and techniques that use protoplasts to study plant responses to both biotic and abiotic stresses and particularly on transient expression assays (TEA), on the use of fluorescence probes and on patch-clamp assays (Figure 1 for an overview). We will also enlighten and discuss the advantages and the limitations of protoplasts as a proxy for whole tissues or plants.

## Protoplasts As Tools in Biomolecular Studies

Protoplasts represent cell populations that are adapted for synchronous pharmacological and biochemical treatments and efficient genetic transformation (Sheen, 2001; Yoo et al., 2007; Xing and Wang, 2015). As TEAs in protoplasts can provide results in less than 36h (Yoo et al., 2007), they are a useful system to investigate early and transient events in plants during stress response at the biomolecular scale. TEAs are performed by isolating protoplasts from plant tissues, transfecting them in the presence of polyethylene glycol and calcium (Yoo et al., 2007; Lin et al., 2014) or through electroporation (Miao and Jiang, 2007) and incubating them for 2–24h. They have been developed in several plant species such as *Arabidopsis thaliana* (Asai et al., 2002; Boudsocq et al., 2004, 2010; Bethke et al., 2009; Li et al., 2019), maize (Kovtun et al., 1998), rice (Takai et al., 2007; Wang et al., 2014; Liu et al., 2018), barley (Saur et al., 2019), wheat (Hahn et al., 2020), strawberry (Gou et al., 2020), banana (Wu et al., 2020b), and rubber tree (Zhang et al., 2016). This system can be used for high-throughput analysis of plant signaling pathways and regulatory mechanisms (Figure 1).

### Functional Screening of Proteins

Plant signaling involves several large protein families which contain many members. For example, in *Arabidopsis*, mitogen-activated protein kinase (MAPK), MAPK kinase (MAPKK), and MAPKK kinase families contain 20, 10, and 60 members, respectively (Bigeard and Hirt, 2018), calcium-dependent protein kinase (CDPK) family has 34 members (Boudsocq et al., 2010), and TFs families such as MYB TFs, WRKY TFs, and basic leucine zipper transcription TFs comprise more than 176, 75, and 78 members, respectively (Dubos et al., 2010; Dröge-Laser et al., 2018; Wani et al., 2021). However, depending on the type of stress, the proteins involved in the signaling cascade may differ and a better understanding of plant defense mechanisms is therefore linked to the identification of its signaling components.

By avoiding time-consuming whole plant transformation, protoplasts offer a useful system to perform functional genomic screen among a group of proteins and determine which of them are able to activate defense genes. The screening is performed with reporter gene assay comprising a number of TEAs equal to the number of proteins or combination of proteins tested. In each TEA, protoplasts are transfected with 2 or 3 vectors simultaneously. One vector expresses the gene coding a protein of interest and has therefore a different sequence in each TEA. Then, a reporter gene often associated with a control gene, both constant between TEAs, can be expressed either in one (Hellens et al., 2005; Liu et al., 2018) or two different vectors (Asai et al., 2002; Boudsocq et al., 2010). The reporter gene is under a stress-inducible promoter that allows the detection of defense gene induction, while the control gene is under a constitutive promoter and allows the normalization of the reporter gene activity by taking into account experimental variation such as differences in cell number, in cell viability, and transformation efficiency (Kovtun et al., 1998). The firefly luciferase or the GFP gene is commonly used as reporter gene, while the ß-glucuronidase (GUS) or the *Renilla* luciferase gene is often used as control gene (Kovtun et al., 1998; Sheen, 2001; Asai et al., 2002; Yoo et al., 2007; Wehner et al., 2011; Thévenin et al., 2012; Liu et al., 2018). The choice of the stress-inducible promoter represents the main limitation of the reporter gene assay as it has to be determined either based on the literature, or by detecting gene activation with PCR (Asai et al., 2002; Boudsocq et al., 2010; Chen et al., 2010). Finally, using the microtiter plate-based protoplast transactivation (PTA) system established by Wehner et al. (2011), high-throughput functional genomic screening can be performed to rapidly analyze up to 96 proteins.

Using this approach, screening of protein kinase families, such as MAPK and CDPK, and TFs has been performed to identify the one(s) involved in plant response to a specific biotic (Asai et al., 2002; Boudsocq et al., 2010; Sheikh et al., 2016) or abiotic stress (Chen et al., 2010; Wehner et al., 2011). When combined with RT-qPCR analysis, TEAs in protoplasts can also reveal potential synergic or antagonist effect between signaling pathways of signaling proteins (Asai et al., 2002; Boudsocq et al., 2010). Moreover, the use of vectors expressing structural variants of the protein of interest could evidence structural motifs compulsory for the signaling function of proteins (Mueller et al., 2012; Pecher et al., 2014). Such variants have provided clues on how allele selection plays a role in climate adaptation of some subspecies (Liu et al., 2018). Reporter gene assays in *Arabidopsis* protoplasts have also demonstrated the complex regulation between catalytic and regulatory subunit of sucrose non-fermenting1-related Kinase1 (SnRK1), involved in metabolic stress response and development (Ramon et al., 2019).

### Protein Location and Trafficking

Besides the functional role of proteins in gene regulation, TEA can also provide information on their subcellular locations and dynamics (i.e., their mobility) into the cell when protoplasts are expressing both the studied protein fused with a fluorescent one, such as YFP, GFP, CFP, or mCherry, and a fluorescent marker specific of a cellular compartment. To that end, several markers have been developed to mark specifically plant organelles (Nelson et al., 2007; Zhang et al., 2021), and their diversity for the different organelles has been recently reviewed (Zhu et al., 2020). These information may help to elucidate protein function (Nelson et al., 2007), since TFs are expected to be found in the nucleus (Asai et al., 2002; Sheikh et al., 2016; Moon et al., 2019), protein receptors in the PM (Li et al., 2017; Liu et al., 2017; Pham et al., 2020), and proteins with a more versatile function can be found both in the cytosol and in cellular organelles (Boudsocq et al., 2010). Fluorescent-tagged proteins in protoplasts have also been used to investigate the influence of the CW on PM protein dynamics (Daněk et al., 2020), the importance of membrane lipid composition in protein cell location (Nagano et al., 2016), and protein trafficking during signaling (Underwood et al., 2017; Menzel et al., 2019). TEA in protoplasts can also bring additional information on protein trafficking with secretion assays to identify and study vacuolar sorting receptor (daSilva et al., 2005; Shen et al., 2013) or signal peptide (Denecke et al., 1990) involved in the regulation of secretory pathways in plant.

### Detection of Protein–Protein Interaction

The study of protein–protein interaction (PPI) through TEAs in protoplasts can also bring crucial information to decipher kinase signaling in plant cells (Pecher et al., 2014; Cheng et al., 2015; Liu et al., 2017; Ye et al., 2019; Li et al., 2020; Takahashi et al., 2020), the activation and interaction of TFs (Pecher et al., 2014; Liu et al., 2018; Ye et al., 2019), or even the interaction between immune receptors and co-receptors (Halter et al., 2014; Yeh et al., 2015; Fliegmann et al., 2016; Gong et al., 2019; Li et al., 2019).

**Figure 2:**
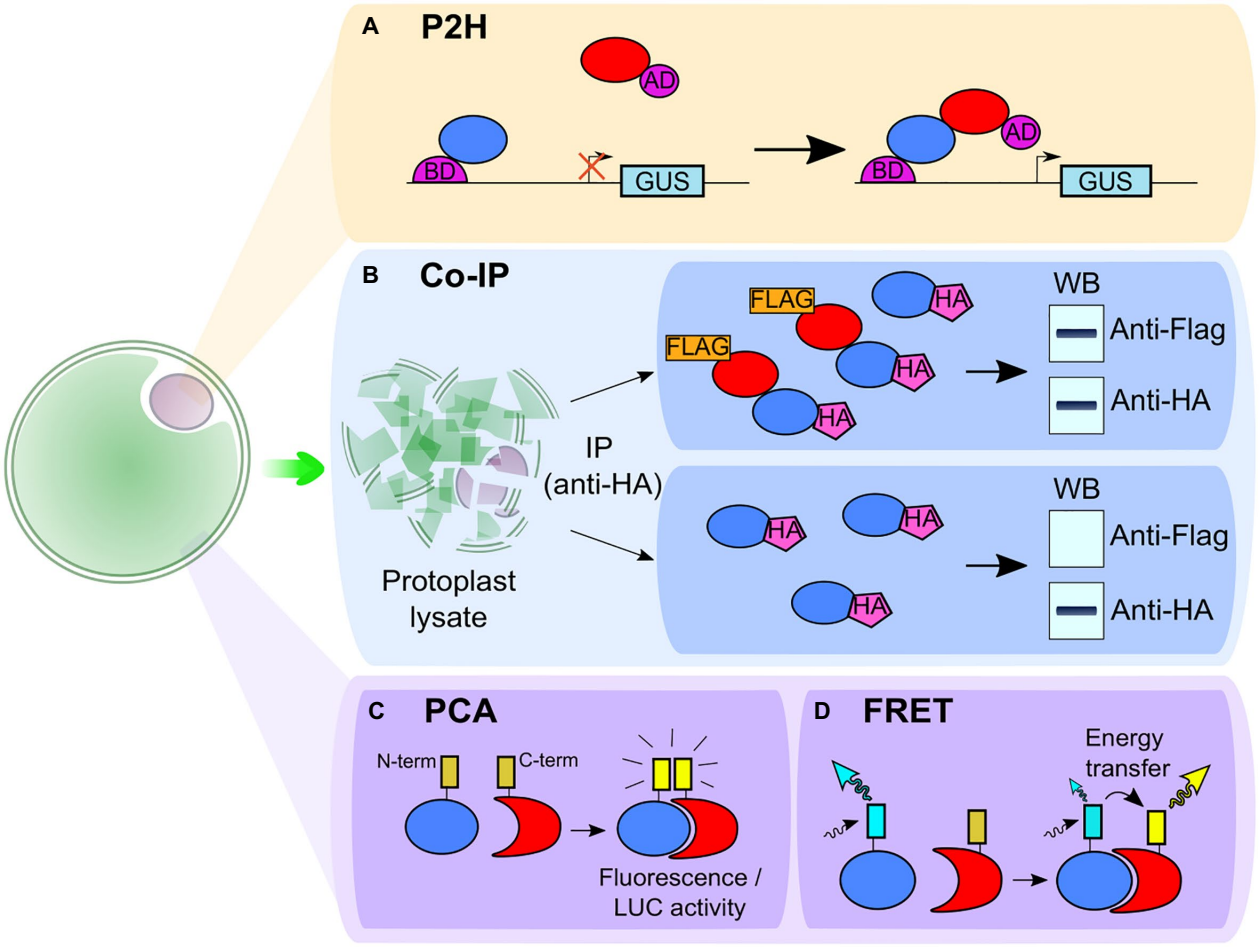
Schematic representation of techniques associated with protoplasts to study protein–protein interaction. **(A)** In the protoplast two-hybrid (P2H) system, the protoplasts are transformed with the GAL4-UAS4:GUS reporter plasmid and the two putative interacting partners are fused to the binding domain (BD) and the activation domain (AD) of the transcriptional activator Gal4. An interaction between the partners leads to the transcription of the glucuronidase (GUS) gene and to a higher GUS activity. **(B)** In co-immunoprecipitation (co-IP), the two putative interacting partners are fused to two different epitopes [e.g., hemagglutinin (HA)-tag and FLAG-tag]. The proteins are then extracted from protoplast lysate with a co-IP using one of the two tags (here, the HA-tag). If the proteins are interacting, both HA-tagged and FLAG-tagged proteins are extracted which can be observed with a western blot (WB; upper box). If the proteins do not interact, only the HA-tag is detected in the western blot (bottom box). **(C)** In protein complementation assay (PCA), such as bimolecular fluorescence complementation (BiFC) or split luciferase complementation (SLC) assay, two fragments (the carboxy-terminal and the amino-terminal parts) of a reporter protein are fused to two putative interacting partners. In BiFC, the fragments come from a fluorescent protein and in SLC, from a luciferase enzyme. The interaction between partners leads to the association between the N- and C-ter fragments and restores fluorescence (for BiFC) or luciferase activity detected in the presence of luciferin (for SLC). **(D)** In Förster Resonance Energy Transfer (FRET), each of the two putative interacting partners is fused with one different fluorophore, either a donor or an acceptor. When the partners are interacting, the donor can transfer its energy of excitation to the acceptor, inducing a change in fluorescence intensity of the fluorophores and in the fluorescence lifetime of the donor.

The yeast two-hybrid (Y2H) is a widely used high-throughput method to detect putative PPI and screen a broad range of interactions between proteins (Pecher et al., 2014; Wang et al., 2014; Liu et al., 2017, 2018; Gong et al., 2019; Ye et al., 2019). However, the physiology of the yeast cell differs from that of the plant cell. To get a system more representative of plant cell physiology, a protoplast two-hybrid (P2H) system has been developed. This approach studies PPI by transferring the GAL4-based two-hybrid system into plant protoplasts instead of yeast cells (Figure 2A; Ehlert et al., 2006; Iven et al., 2010). Hence, the P2H system identifies PPI between two proteins by fusing one of them with the binding domain (BD) and the second protein with the activation domain (AD) of the transcriptional activator Gal4. With the use of GAL4-UAS4:GUS reporter plasmid, the PPI is detected when a higher GUS activity is observed. When studying interaction between leucine zipper TFs, this method was able to detect some weak interactions not detected in Y2H system, suggesting that P2H studies may be more representative of *in planta* conditions than Y2H (Ehlert et al., 2006; Xing and Wang, 2015). P2H has also been used to analyze PPI involved in the regulation of heat shock response in *Arabidopsis* (Hsu et al., 2010) and in auxin signaling in tobacco (Böttner et al., 2009). Furthermore, in a similar way as it was developed for functional genomic screening, a high-throughput PPI screening can be performed with the combination of P2H and a microtiter plate-based system (Wehner et al., 2011).

Nevertheless, since both Y2H and P2H studies are performed in the nucleus, they are therefore possibly limited to specific classes of proteins such as TFs (Ehlert et al., 2006) and complementary approaches using TEA in protoplasts should be considered to confirm PPI *in planta*. These additional techniques comprise co-immunoprecipitation assays (co-IP; Figure 2B; Li et al., 2019; Ye et al., 2019), protein complementation assays (PCA), including bimolecular fluorescence complementation (BiFC; Pecher et al., 2014; Liu et al., 2018; Takahashi et al., 2020; Yang et al., 2020) and split luciferase complementation (SLC; Figure 2C; Cheng et al., 2015; Gong et al., 2019) and Förster Resonance Energy Transfer (FRET) experiments (Figure 2D; Halter et al., 2014; Fliegmann et al., 2016).

To study PPI with co-IP (Figure 2B), protoplasts are transformed with two vectors, each containing one of the proteins of interest fused with a different epitope, such as the hemagglutinin (HA) tag or the FLAG tag (Cheng et al., 2015; Li et al., 2019). The protoplasts are then lysed, and the proteins are immunoprecipitated using one of the two epitopes. The resulting extract is then analyzed by western blot to detect the second epitope and confirm the PPI. This approach has been used in *Arabidopsis* protoplasts to demonstrate the association of the receptor-like kinase (RLK) BAK1 with a calcium channel when studying plant cell death (Yu et al., 2019). Still in *Arabidopsis*, it highlighted the negative effect of the RLK NIK1, involved in antiviral immunity, on bacterial immunity by showing its impact on the formation of the complex between FLS2 and its co-receptor BAK1, paramount in the sensing of the bacterial PAMP flagellin22 (flg22; Li et al., 2019). Co-IP experiments on protoplasts also evidenced the importance of ubiquitination of another RLK, BIK1, for plant immune response regulation (Ma et al., 2020). Nevertheless, co-IP does not provide spatial nor temporal information on PPI. Besides, it is an *in vitro* approach and the lysis process may disrupt weak interaction or induce interaction between proteins that would never be brought together under physiological conditions (Struk et al., 2019). Hence, if the PPI studied is transient, other *in vivo* approaches should be considered such as PCA or FRET (Cui et al., 2019; Struk et al., 2019; Takahashi et al., 2020).

In PCA (Figure 2C), one of the studied proteins is fused with the amino-terminal part and the other one with the carboxy-terminal part of a fluorescent protein, such as YFP or mCherry, for BiFC (Pecher et al., 2014; Cheng et al., 2015; Li et al., 2020) or a luciferase enzyme, such as the firefly luciferase, for SLC (Chen et al., 2008; Cheng et al., 2015; Gong et al., 2019). In BiFC, when the two proteins interact, the combination of the two parts of the fluorescent protein restores the fluorescence enabling the visualization and the spatial location of protein complexes. In SLC, the interaction of the two proteins restores the luciferase activity which can be detected in the presence of luciferin by the measurement of chemiluminescence. Thanks to PCA on protoplasts, information on *in vivo* PPI can be obtained, but both techniques have their specificities. With BiFC, the location of both long-standing and transient PPI can be observed, while the high background signal observed with SLC prevents such observation (Fujikawa and Kato, 2007; Cui et al., 2019). In *Arabidopsis*, BiFC has shown the PM location of the interaction between proteins involved in stress response. For instance, it evidenced the interplay between the ATP-recognition receptor DORN1 and the NADPH oxidase RBOHD, involved in ROS production and stomatal closure (Chen et al., 2017). It also showed the nitrate-sensing mechanism where transceptor NRT1.1, that acts as nitrate transporter and sensor, interacts with the calcium channel CNGC15 (Wang et al., 2021). However, the irreversible recombination of fluorescent proteins used in BiFC limits its ability to study PPI dynamics and SLC offers a better alternative in that regard (Kerppola, 2006; Kudla and Bock, 2016; Cui et al., 2019; Struk et al., 2019). Indeed, the reversibility of luciferase recombination allows detection of both the association and dissociation of two proteins in less than 1min following treatment (Li et al., 2011; Wang et al., 2020b).

Another way to study PPI dynamics and location *in vivo* with protoplasts is the use of FRET. Here, two putative interacting partners are fused with a fluorophore (Halter et al., 2014; Fliegmann et al., 2016; Rios et al., 2017; Long et al., 2018). One partner is fused with a donor fluorophore, while an acceptor fluorophore is fused to the putative interacting partner (Figure 2D). The donor fluorophore displays an emission spectrum that overlaps with the excitation spectrum of the acceptor fluorophore. When the proteins interact, it brings the donor in close proximity to the acceptor allowing a transfer of energy from the first fluorophore to the second. This leads to a decrease in fluorescence intensity and lifetime of the donor concomitant with an increase in fluorescence intensity of the acceptor. As FRET is based on a remote interaction and not a physical interaction between the tags of the proteins of interest, this approach allows the study of PPI dynamics with information on protein location. It has, for instance, been used in *Arabidopsis* protoplasts to show the early disruption of the interaction between the ethylene factor ERF104 and MAPK6 following treatment with flg22 (Bethke et al., 2009). The implementation of FRET analysis first requires an optimization of the labeling condition. In this regard, TEAs in protoplast represent a convenient tool to test a large number of FRET pair combinations before transposing it to whole plants or tissues (Long et al., 2018). Nevertheless, FRET measurements require a high accumulation level of the protein of interest and advanced equipment to detect the signal, explaining its limited use in PPI studies (Cui et al., 2019; Struk et al., 2019).

In summary, TEAs in protoplasts associated with the aforementioned techniques provide useful tools to study PPI in plant cells. Each technique has its own characteristics and limitations hence why a complementary use of several of them should be envisaged to get a reliable and comprehensive view of PPI. PPI studies are, however, not restricted to protoplasts, and readers interested in PPI analysis in other systems may refer to recent reviews (Cui et al., 2019; Struk et al., 2019).

### Detection of Kinase Activity and Protein Phosphorylation

Following the identification of PPI, one could be interested in understanding its consequences, such as the activation of kinases or protein phosphorylation. To that end, crude or immunoprecipitated protein extracts are collected from lysates of protoplasts or plant seedlings having undergone biotic or abiotic stress. Compared to experiments in plant seedlings which require mutant generation, protoplasts transiently expressing the studied protein(s) provide a high-throughput system to perform explorations as well as hypothesis-driven tests as results can be obtained in a few days (Yoo et al., 2007). For instance, *Arabidopsis* protoplasts have been used to study flg22-induced phosphorylation of the RLK BIK1 (Li et al., 2019) and investigate the importance of amino acid residue for protein phosphorylation in PAMPs-triggered immunity (Menzel et al., 2019) and in cold stress (Ye et al., 2019). The assessment of kinase activation is then performed either by the detection of the kinase activity through the phosphorylation of kinase substrate or by detecting phosphorylated kinases as their activation is linked to their phosphorylation state.

To detect kinase substrate phosphorylation, proteins extracted from protoplasts are incubated with the radioactive marker γ[^32^P]ATP and a substrate, which can be a protein, such as a histone of myelin basic (Asai et al., 2002; Boudsocq et al., 2004, 2010; Liu et al., 2017), a kinase, such as MAPK for MAPKK (Asai et al., 2002; Wang et al., 2014), or even a lipid (Menzel et al., 2019). Once incubated, the kinase activity is determined by measuring the incorporation of the radioactive marker into the kinase substrate. To avoid the use of radioisotopes, an alternative method to measure the phosphorylation of kinase substrate has been developed using a phosphate-binding tag (Phos-Tag) assay (see below; Kinoshita et al., 2006).

Finally, protein phosphorylation can be detected either with specific antibody or by observing mobility shift of proteins with SDS-PAGE (Pecher et al., 2014; Li et al., 2019; Yu et al., 2019; Ma et al., 2020). To perform immunodetection, crude protein extract is analyzed by western blot with a primary antibody recognizing phosphorylated amino acids (Gong et al., 2019; Li et al., 2019) or motifs such as dual phosphorylation specific to active MAPKs detected with anti-pERK antibody (Cheng et al., 2015; Zhang et al., 2016; Gong et al., 2019). For the mobility shift assay, Phos-Tag can be added into the SDS-gel to improve the separation between non-phosphorylated and phosphorylated proteins (Kinoshita et al., 2006; Kinoshita-Kikuta et al., 2007; Bekesová et al., 2015). Thanks to protoplast-associated Phos-Tag mobility shift assay, the phosphorylation of kinases (Bi et al., 2018; Menzel et al., 2019), TFs (Ye et al., 2019), or other proteins (Liu et al., 2017) involved in the signaling process in biotic and abiotic stress has been detected. Hence, while being an alternative to radioisotopes, Phos-Tag assays are also a suitable alternative to antibody recognizing phosphorylated proteins, which are costly or even not always commercially available, to detect protein phosphorylation and kinase activation (Bekesová et al., 2015; Kinoshita et al., 2015). Finally, to confirm that the mobility shift observed is due to phosphorylation, treatment with phosphatase to abrogate the mobility shift is often performed (Pecher et al., 2014; Liu et al., 2017; Bi et al., 2018; Li et al., 2019). Additionally, TEA in protoplasts can also provide information on the consequence of phosphorylation such as the degradation of calcium channels (Yu et al., 2019) or TFs that regulate stress-related genes (Sheikh et al., 2016; Liu et al., 2017).

### Complementarity of Biomolecular Assays Performed on Protoplasts and Whole Cells

As presented above, many biomolecular assays have been developed with protoplasts to decipher plant signaling mechanisms in biotic or abiotic stress conditions. All these different types of assays offer a useful toolbox to analyze plant responses and get new insights to better understand the signaling cascade in plants, starting from the perception by a protein receptor to the activation of TFs and genes, passing by the kinase signaling cascade.

The use of these tools is not restricted to protoplasts, and TEA can be performed directly in plant tissues using particle bombardment or *Agrobacterium* infiltration (Cheng et al., 2015; Liu et al., 2017, 2018; Bi et al., 2018; Pham et al., 2020; Takahashi et al., 2020). The latter is used either to transform only specific plant tissue or to produce transgenic plant lines constitutively expressing the gene of interest (Wu et al., 2014a; Sharma et al., 2018). Nevertheless, all these approaches present advantages and limitations. Therefore, TEAs performed in protoplasts are complementary to TEAs performed in intact plant tissues and constitutive expression in mutant plants (Denecke et al., 2012; Sharma et al., 2018). Indeed, protoplasts are obtained from the digestion of tissues containing a mixture of differentiated cell types that can display different locations of specific proteins (Faraco et al., 2011). Even though some protocols exist to isolate protoplasts of specific cell types such as guard cell (Zhao et al., 2019), aleurone layer cell (Daneri-Castro and Roberts, 2016), or from various root tissues (Demidchik et al., 2003), the complementary use of transgenic plants is recommended if a tissue-specific behavior of the process studied is anticipated (Sharma et al., 2018). Instead, if no tissue-specificity is expected, protoplasts offer a valuable model to study physiological processes as it is less time-consuming to obtain than transgenic plant and can be performed in a broad range of plant species, contrary to agroinfiltration in leaves that are mainly restricted to the plant host *Nicotiana benthamiana* (Sharma et al., 2018). Furthermore, in agroinfiltration experiments, the moment when the gene transfer occurs is not well defined. On the contrary, with protoplasts transformation, the moment where the DNA transfer happens is well known and gene products can be detected as early as 4h after gene transfer (Denecke et al., 2012). Hence, protoplasts are a useful system to perform time-course experiment of gene expression (Babu et al., 2008), which are more difficult to carry out in infiltrated cells (Denecke et al., 2012).

Even though TEA in protoplasts or intact cells can bring precious findings, some cautions must be taken when using these tools. The experiment must be carefully designed to avoid overexpression artifacts which can lead to artificial cytosolic location, or even aggregation of the protein (Sharma et al., 2018). This can be done by adapting the amount of DNA plasmid used for transformation or the incubation time of protoplasts for gene expression, usually less than 24h, to obtain low-expressing protoplasts for experimental purpose (Yoo et al., 2007; Denecke et al., 2012). Moreover, the enzymatic CW digestion performed to isolate protoplasts may stress the cell which could alter the expression levels of some genes (Birnbaum et al., 2003; Takai et al., 2007; Jeworutzki et al., 2010). As an example, in a screen of more than 22,000 *Arabidopsis* genes, 356 were found to be induced at least twice more by the CW digestion (Birnbaum et al., 2003) and some flagellin-inducible genes have also shown higher expression following protoplast isolation in rice (Takai et al., 2007). Such induction of genes in protoplasts may alter cell responses to stimulus such as the activation of ion channels (Jeworutzki et al., 2010). In addition to altered gene expression, protoplast isolation can change the sensitivity of cell enzymes to its inhibitor, as shown for phosphoenolpyruvate carboxylase regarding malate inhibition (Petropoulou et al., 1990). It is therefore important to assess that the biological response in protoplasts is not disturbed compared to intact plant cells. Gene induction or protein accumulation similar to whole plants levels (Asai et al., 2002; Boudsocq et al., 2004; Underwood et al., 2017) and the verification with fluorescent probes of protoplast integrity are possible controls.

## The Versatility of Fluorescent Probes on Protoplasts

With the advent of cell imaging technologies, fluorescence microscopy has been increasingly used for the visual insight it provides. While many probes can be used on plant tissues, autofluorescence and probe specificity have turned out to be an issue. Some dyes also tend to accumulate within the CW microfibrils, tempering with the imaging process (Blachutzik et al., 2012). Fluorescent probes applications on protoplasts appear then as particularly useful since they allow observations at the single cell level without the issues caused by the presence of the CW (Figure 1 for an overview).

### Cell Viability and DNA Damages

Fluorochromes are often used to discriminate between living and dead protoplasts and to assess their viability and the damages they might have suffered. Indeed, protoplast isolation procedures and the culture conditions that follow, may induce cell stress or damage (Neelakandan and Wang, 2012), which should be avoided if one wants to study the effect of biotic and abiotic stresses. One of the most referenced dyes is FDA (fluorescein diacetate), which highlights living cells (Bertini et al., 2019; Sangra et al., 2019; Qiu et al., 2020) or Evan’s blue, which highlights dead ones (Kollárová et al., 2019). Other fluorochromes can be used, such as PI (propidium iodide), DAPI (4'6-diamidino-2-phenylindole, dichloride), and 7-AAD (7-amino-actinomycin D) that do not cross intact PMs. Issues have previously been raised regarding techniques using fluorescence microscopy, as the quantification is linked to the viewer’s perception of fluorescence (Aoyagi, 2011; Badaró Costa et al., 2018). Thus, new automated measurements, such as flow cytometry (FCM; Zhou et al., 2019; González-García et al., 2020) and Muse cell analyzer, a compact FCM, allowing screening and sorting of protoplasts, along with measures on smaller volumes have been developed (Badaró Costa et al., 2018).

DNA damage evaluation is another frequently employed marker to assess protoplast viability or the effect of genotoxicity of environmental pollutants and abiotic stresses on protoplasts. In these procedures, protoplasts are used as a direct source of nuclei to perform gel electrophoresis with ethidium bromide staining in order to detect DNA laddering (Poot-Poot et al., 2016). Identically, single cell gel electrophoresis assay (SCGE), also called Comet assay, allows the study of DNA damage on protoplasts at the single cell or nuclei level (Kuzminsky et al., 2016; Badaró Costa et al., 2018; Choury et al., 2018). While this technique is amply used on animal cell cultures which are easily lysed, the presence of the CW makes it technically difficult to transpose on plant tissue or cell culture. Hence, nuclei isolation through protoplast formation or mechanical destruction of the CW is here preferred (Gichner et al., 2009; Santos et al., 2015; Choury et al., 2018). Finally, DAPI, which has a high affinity for DNA double strand, has also been used to study apoptosis-like cell death and more specifically chromatin condensation and DNA fragmentation in *Brassica napus* leaves (Watanabe et al., 2002).

### Cell Wall Dynamics

The CW has a direct role at the frontline of plant defense along with other chemical and physical barriers such as waxes, hairs, and secondary metabolites (Malinovsky, 2014; Engelsdorf et al., 2018). It also possesses an indirect role in plant defense systems, as during a pathogen invasion, cell wall integrity can be modified, parts of the CW can be broken down and their fragments (referenced as DAMPs) can activate plant immune responses (Souza et al., 2020). As protoplasts are cells deprived of CW, they offer a unique point of view on the complete *de novo* synthesis of the CW by providing an excellent support for visualizing its regeneration dynamics and characterizing the cellular proteins involved in the process (Yokoyama et al., 2016). Although changes in CW composition are often studied through biochemical analyses, histochemical staining with fluorochromes is increasingly used to bring a visual insight on these changes. For instance, calcofluor white is employed to preferentially stain cellulose and aniline blue to stain callose (Yokoyama et al., 2016; Kollárová et al., 2019). Using these probes, it has, for instance, been demonstrated that when cultivated in stressful conditions, cellulose microfibrils were not deposited on the surface of white birch protoplasts and only callose deposition could be observed (Tagawa and Kondo, 2018; Tagawa et al., 2019). Calcofluor white has also been used to study the deleterious effect of cadmium on maize protoplast CW regeneration (Kollárová et al., 2019). Another method also emerged using S4B (Pontamine Fast Scarlet 4 BS) in combination with spinning disk confocal microscopy to stain cellulose patterning on living cells. As calcofluor has toxic properties that might injure cells, this method appears to be more suited to real-time imaging of living protoplasts (Anderson et al., 2010; Yokoyama et al., 2016; Kuki et al., 2017).

Similarly, CW components and callose deposition are known to block the migration of trace metals within cells, such as aluminum which binds to calcium pectate in the CW (Lee et al., 2001). Therefore, protoplasts are often combined with specific fluorochromes to study the effects and uptake of trace metals directly on cells (Krzesłowska, 2011). For instance, Leadmium was used to visualize the uptake of cadmium by protoplasts and its deleterious effects on CW regeneration of wheat (Greger et al., 2016) and maize (Kollárová et al., 2019). Similarly, morin was used to study aluminum toxicity on coffee protoplasts, along with DAPI to monitor its localization into their nuclei (Poot-Poot et al., 2016). It was also used to examine its toxicity on root protoplasts of transgenic camelina (Park et al., 2017).

### Plasma Membrane Dynamics

Along with the CW, the PM also plays a major role in plant resistance to both biotic and abiotic stresses. Whether it is by regulation of ion exchanges, perception of PAMPs/MAMPs/DAMPs, or signal transduction, both lipids and proteins of the PM are key players in its physiological function (Lim et al., 2017; Mamode Cassim et al., 2019; Schellenberger et al., 2019; Huby et al., 2020; Saijo and Loo, 2020). Moreover, following the CW, the PM is the first point of contact between plant cells and pathogens and many proteins involved in plant defense are embedded in it. More specifically, the dynamic between membrane microdomains, which are highly ordered domains rich in sphingolipids and sterols, and the stress-related proteins they harbor is crucial for immunity (Gronnier et al., 2016, 2018; Nagano et al., 2016; Mamode Cassim et al., 2019; Huby et al., 2020).

The absence of CW makes possible the accurate visualization of events at the protoplast PM using fluorescent probes. However, while a lot of probes exist to study lipid organization and dynamics into artificial model membranes which are deprived of proteins, they often cannot be directly applied to living cell and protoplast PMs which are far more complex and require deep protocol adaptations in terms of concentration and incubation time (Klymchenko and Kreder, 2014). Every probe will have its specificities and are used by themselves or combined. For instance, FM4-64 [N-(3-Triethylammoniumpropyl)-4-(6-(4-(Diethylamino) Phenyl) Hexatrienyl) Pyridinium Dibromide] and LRB-PE (Lissamine Rhodamine B-Phosphoethanolamine) have been employed to specifically stain phospholipid enriched areas of protoplast PM and BD-SM (Bodipy Sphingomyelin FL C_12_) has been used to stain sphingolipid enriched domains (Blachutzik et al., 2012). FM4-64 and BD-SM were also used in combination with FRAP (fluorescence recovery after photobleaching) experiments to visualize lipid redistribution. The identification of ordered and disordered regions of the PM is also possible with the solvatochromic dyes di-4-ANEPPDHQ and laurdan that show a shift in emission wavelength when lipids undergo phase transition from gel to fluid state (Blachutzik et al., 2012; Klymchenko, 2017). Di-4-ANEPPDHQ has notably been used on protoplasts from rice transgenic plants that lack fatty acid hydroxylase 1 and 2 (FAH1/2), enzymes responsible for the formation of 2-hydroxy sphingolipids (2-OH-SL), precursors of glycosylinositol phosphorylceramides (GIPC), that are both located at the PM in *Arabidopsis*. They demonstrated that a disordered PM was concomitant with a lower amount of 2-OH-SL which gave rise to an increased sensibility to rice blast fungus infection (Nagano et al., 2016). Di-4-ANEPPDHQ has also been used in *Arabidopsis* FAH1/2 mutants, to show a lower order of the PM compared to the wild type, suggesting an altered PM organization when its content in GIPC is low (Lenarčič et al., 2017).

While there are many advantages to use fluorescent probes directly on protoplasts, its PM remains an active, dynamic structure, which can cause issues. It has been reported that some probes could be internalized in the cytoplasm, such as DiIC12 (1,1'-Didodecyl-3,3,3',3'-Tetramethylindocarbocyanine Perchlorate) and DiIC18 (1,1'-Dioctadecyl-3,3,3',3'-Tetramethylindodicarbocyanine-5,5'-Disulfonic Acid), which stains phospholipids, leading to a decrease in fluorescence in the PM (Blachutzik et al., 2012). By using calcofluor and di-4-ANEPPDHQ on tobacco protoplasts, it has been shown that the absence of a CW does not affect the organization of PM-ordered domains (Grosjean et al., 2018), suggesting that the PM microdomain functions of a protoplast remain highly similar to that of an intact tissue. However, in *Arabidopsis*, FRAP analysis proved that the removal of the CW increased the overall dynamics and mobility of the PM proteins (Martinière et al., 2012), including proteins involved in response to extracellular stimuli flotilin2 proteins (AtFLOT2) and hypersensitive induced reaction proteins (AtHIR1; Daněk et al., 2020).

### Detection of Early Stress Signaling Events

Fluorescent probes can also be useful to detect specific early stress signaling events like ROS production and ions fluxes. For instance, in *Arabidopsis*, the molecular probe ContPY1 was used to detect the intracellular accumulation of a ROS, hydrogen peroxide (H_2_O_2_) in response to the elicitor COS-OGA. The comparison between protoplasts and cell suspensions evidenced the relative contribution of CW peroxidases and membrane dehydrogenases to H_2_O_2_ production (Ledoux et al., 2014). On maize, the Amplex red reagent, which reacts with H_2_O_2_ to produce the highly fluorescent resorufin, and the rhodamine dye DHR123 (Dihydrorhodamine 123) were used to measure ROS in both organelles and protoplasts and link their quantities to DNA damage in developing mitochondria and plastids (Tripathi et al., 2020).

Similarly to ROS production, ion fluxes can be easily studied with fluorescent probes associated with protoplasts. For instance, K^+^ efflux was monitored with the fluorescent probe PBFI-AM (Potassium-Binding Benzofuran Isophthalate Acetoxymethyl ester) and cytosol acidification with the pH-sensitive probe BCECF-AM[2',7-Bis-(2-Caboxyethyl)-5-(and-6)-Carboxyfluorescein Acetoxymethyl ester] in wheat and rice protoplasts to study anoxia-induced events (Yemelyanov et al., 2020). Furthermore, protoplasts loaded with the probe SBFI-AM (Sodium Binding Benzofuran Isophthalate Acetoxymethyl ester) were used to study salt stress on wheat. They helped to demonstrate that the application of a moderate amount of K^+^ was concomitant with a decrease in cytosolic Na^+^ alleviating its toxic effects on cells (Gul et al., 2019). Regarding Ca^2+^ fluxes, their induction has been monitored in elicited protoplasts expressing the genetically encoded reporter system aequorin, a bioluminescent protein (Maintz et al., 2014). This technique can, however, be lengthy, especially for slow growing plants such as fruit trees since it requires plant transformation (Qiu et al., 2020). So small dyes like fluo-8/AM, fluo-4/AM (fluo-8/4 acetoxymethylester) and rhod-2/AM (rhod-2 acetoxymethylester) can be preferred. These molecules are flexible, rapid, and non-cytotoxic. They have been used for calcium imaging on protoplasts of “Fuji” apples (Qiu et al., 2020). Fluo-4/AM has also been used with FCM and confocal microscopy on rice protoplasts to evaluate ceramide-induced programmed cell death (Zhang et al., 2020). While there are many advantages to fluorescent probes to study ion fluxes in protoplasts, there are still some limitations such as the commercial availability of probe sensitive to anions.

## Protoplasts and Patch-Clamp Electrophysiology

Complementary to fluorescent probes, plant ion fluxes can be studied using patch-clamp electrophysiology that measures ion currents flowing through a membrane (Demidchik et al., 2006; Elzenga, 2012). This technique is a powerful tool to identify and characterize ion channel and non-channel proteins, such as H^+^-ATPases, present in biological membranes (Demidchik et al., 2006; Elzenga, 2012; Hamilton et al., 2015). To measure the ionic current with patch clamp, a high resistance contact, the so-called gigaOhm seal, has to be performed between a glass micropipette and a patch of a membrane containing the ion transporter of interest (Demidchik et al., 2006; Elzenga, 2012). The access to a CW-deprived plant cell is particularly important to measure PM ionic current (Elzenga, 2012). Hence, protoplasts are the model of choice to perform patch-clamp electrophysiology on plant.

Four patch-clamp configurations exist, and readers interested in this technique may refer to previous reviews for more details on their specificities (Demidchik et al., 2006; Elzenga, 2012). This technique has provided important insights in the understanding of anion channels involved in immunity (Zheng et al., 2018; Chan et al., 2020) and ABA signaling during osmotic stress (Takahashi et al., 2020). It also contributed to a better comprehension of mechanoperception in plant (Nakagawa et al., 2007; Haswell et al., 2008), potassium and calcium fluxes involved in salt stress (Fuchs et al., 2005; Liya et al., 2012). It also helped to elucidate calcium fluxes involved in H_2_O_2_ perception (Demidchik et al., 2007; Tian et al., 2019; Wu et al., 2020a), in extracellular ATP perception (Demidchik et al., 2009), in cold stress (Carpaneto et al., 2007), and in stomatal immunity (Yekondi et al., 2018).

Patch-clamp electrophysiology is the gold standard technique to study ion channels and fluxes even though the process to isolate the cell and remove its CW can be considered a limitation (Demidchik et al., 2006; Hamilton et al., 2015). However, combination of patch clamp with other electrophysiological or physiological techniques using intact plants such as microelectrode ion flux estimation or the use of fluorescent probes can be considered to improve the robustness of the results (Demidchik et al., 2006; Hamilton et al., 2015; Demidchik, 2018).

## Challenges and Future Perspectives

The current and upcoming rise of pests, diseases, and changes of agricultural practices caused by environmental perturbations will put an increasing pressure on agricultural productivity. This will require specific tools allowing fast, high throughput, or even automated systems, to provide reliable and efficient solutions for crop and genetic engineering.

**Figure 3:**
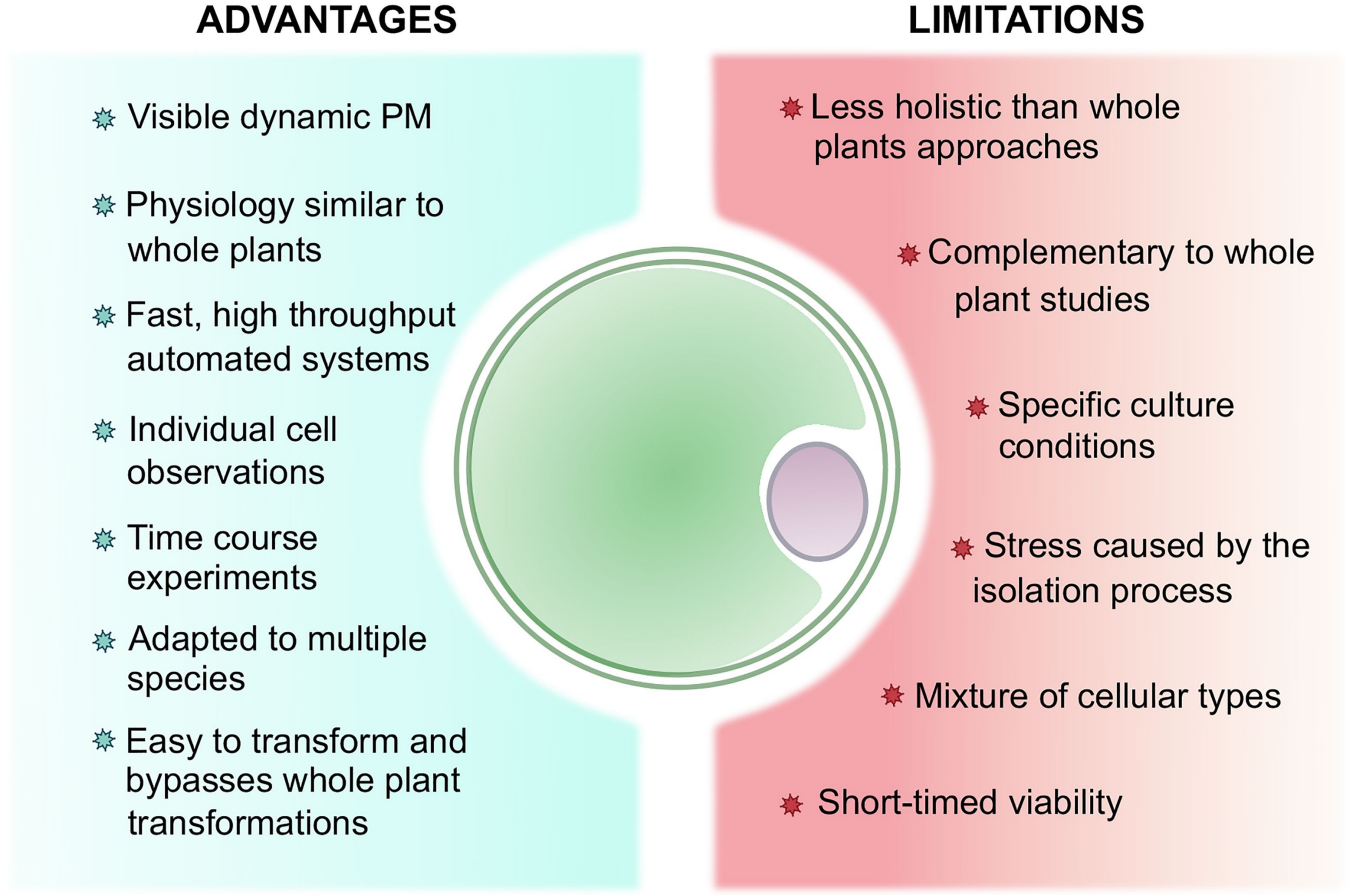
Summary of the advantages and limitations of working with protoplasts to study plant stress perception and response comparatively to whole plants.

With the advent of quick and reliable transformation and microscopy methods, protoplasts arise as useful and powerful tools for a wide range of stress-related studies (Figure 1). We have argued in this review that the use of protoplasts could turn out to be both an advantage and a limitation (Figure 3). While it has been previously stated that protoplasts maintain a similar physiological cellular activity to whole plants (Sheen, 2001; Wang et al., 2020a; Shaw et al., 2021), they ultimately serve as proxy to whole plants studies, implying that complementary experiments are often necessary to connect the phenomena observed on protoplasts to plants. Nonetheless, the single cell level allows for specific, rapid, and high-throughput analysis along with time-course experiments. In addition, even though protoplasts isolation and maintenance require specific conditions that can eventually cause stress, these techniques are improving for a wide range of species or organs (Sangra et al., 2019; Shan et al., 2019; Davis et al., 2020). Furthermore, their formation is still deemed necessary to bypass time-consuming plant culture and whole plant transformation, especially for recalcitrant species or plants with a long reproductive cycle (Du and Bao, 2005). Moreover, somatic hybridization mediated by protoplast fusion has been employed to circumvent sexual incompatibility encountered in plant breeding (Watanabe et al., 2002; Du and Bao, 2005).

Protoplasts possess multiple assets for the upcoming challenges in plant biology. Many technologies employing them are being developed, and these techniques could ultimately facilitate plant stress-related studies. For instance, protoplasts have been employed to assess the efficiency of CRISPR-associated protein 9 (Cas9) mutagenesis, bypassing or preceding stable transformation which can be time-consuming (Lin et al., 2018; Hahn et al., 2020; Guyon-Debast et al., 2021; Nicolia et al., 2021). Another example is the adaptation of efficient and low-cost microfluidic techniques to perform spatiotemporal studies of plant protoplasts physiology during their development (Sakai et al., 2019) and to apprehend the electrical resistance of CW-regenerated protoplasts (Chen, 2020). Similarly, the usefulness of protoplasts for high-throughput RNA sequencing has also been put forward due to its many advantages over traditional RNA-seq. Indeed, protoplasts being single cells, they can give spatiotemporal information on gene dynamic expression in heterogeneous tissues (Li et al., 2021).

Protoplasts also provide a facilitated access to the plant PM, and pioneering studies have proven their crucial role in growing biotechnologies such as nanoparticles. These particles have the ability to passively penetrate the PM, but their use in plants is limited due to the presence of the CW (Torney et al., 2007; Liu et al., 2009; Lew et al., 2018). As they are believed to have the potential to overcome current limitations in plant genetic transformation, their effect on plants and their PMs are increasingly studied (Lew et al., 2018). With their easily accessible PM, protoplasts can therefore help understand the fundamental interactions between nanoparticles and plants, as such knowledge is of paramount importance for nanoenabled agriculture. Protoplasts have, for instance, been used to determine the impact of nanopesticides or nanofertilizers, on plant photosynthesis (Wang et al., 2020a). Likewise, protoplast cultures have been used to study gold nanoparticles uptake by plants as their use in industrial areas leads to their release into the environment, which can cause an invisible danger to the ecosystem (Milewska-Hendel et al., 2019). Furthermore, nanoparticles have been previously used to deliver drugs, imaging agents, and DNA for genetic transformation into protoplasts (Torney et al., 2007).

Concomitantly, protoplasts could be valuable plant PM models to study the perception of bioactive molecules such as elicitors by plant cells. Indeed, they could link data obtained by biophysics studies on biomimetic PM models containing representative lipids (Deleu et al., 2014) with the ones provided by biological assays on living plant cells or tissues with complex dynamic PM and CW. Protoplasts-associated technologies and techniques should help improve our fundamental knowledge on plant perception and response to (a)biotic stresses and hence ultimately contribute to develop reliable and efficient solutions for agriculture.

## Author Contributions

GG, EH, MD, and SD-C designed the outlines of the review and wrote the manuscript with the contribution of SC and MO. All authors contributed to the article and approved the submitted version.

## Funding

This work was supported by the National Fund for Scientific Research (FNRS, Belgium), the SFR condorcet (18ARC107), and the “Fondation Universitaire de Belgique” (Belgian University Foundation). GG, was supported by the Foundation for Training in Industrial and Agricultural Research (FRIA, FNRS, grant 1.E.069.20F), and MD and MO are Senior Research Associates of the FNRS.

## Conflict of Interest

The authors declare that the research was conducted in the absence of any commercial or financial relationships that could be construed as a potential conflict of interest.

## Publisher’s Note

All claims expressed in this article are solely those of the authors and do not necessarily represent those of their affiliated organizations, or those of the publisher, the editors and the reviewers. Any product that may be evaluated in this article, or claim that may be made by its manufacturer, is not guaranteed or endorsed by the publisher.
